# Fire-induced effects on the bioavailability of potentially toxic elements in a polluted agricultural soil: implications for Cr uptake by durum wheat plants

**DOI:** 10.1007/s11356-022-22471-5

**Published:** 2022-08-23

**Authors:** Ida Rascio, Concetta Eliana Gattullo, Carlo Porfido, Ignazio Allegretta, Matteo Spagnuolo, Raphael Tiziani, Silvia Celletti, Stefano Cesco, Tanja Mimmo, Roberto Terzano

**Affiliations:** 1 Department of Soil, Plant and Food Sciences, University of Bari “Aldo Moro”, Via G. Amendola n. 165/A, 70126 Bari, Italy; 2 Faculty of Science and Technology, Free University of Bozen-Bolzano, Piazza Università n. 5, 39100, Bolzano, Italy

**Keywords:** Soil fire, Potentially toxic elements, Polluted soil, Cr(VI), Chromium uptake, Chromium translocation

## Abstract

Fire events can modify the distribution and speciation of potentially toxic elements (PTEs) in soil, especially if they are associated to organic matter (OM). In fact, OM can undergo substantial structural modifications at high temperatures, up to the complete mineralization. The present study aims to investigate the changes of PTEs’ bioavailability to durum wheat (*Triticum durum* Desf.) plants after simulating fire events (up to 300 °C and 500 °C) in an agricultural soil polluted by Cr, Zn, Cu, and Pb. The PTEs’ uptake and allocation in plant tissues were assessed using the RHIZOtest system. After the fire simulations, no evident risk of accumulation and translocation in plants was observed for Zn, Pb, and Cu. Conversely, a high accumulation in roots and a significant translocation to shoots were observed for Cr, which reached concentrations of 829 mg kg^−1^ in roots and 52 mg kg^−1^ in shoots at 500 °C. Additional experimental evidence suggested that Cr was taken up by plants grown on heated soils as Cr(VI). Once acquired by roots, only a small part of Cr (up to 6%) was translocated to shoots where it was likely present as mobile forms, as evidenced by micro X-ray fluorescence (µ-XRF) analyses. Overall, the results obtained provide evidence that the high temperatures occurring during fire events can increase the mobility and bioavailability of certain PTEs transforming apparently safe environments into potentially dangerous sources of pollution. These processes can ultimately affect the human health through the food chain transfer of PTEs or their migration into surface water and groundwater.

**Supplementary Information:**

The online version contains supplementary material available at: 10.1007/s11356-022-22471-5.

## Introduction

Soil contamination by potentially toxic elements (PTEs) increasingly affects a large number of agricultural areas worldwide, causing deleterious effects on crop yield and food safety. Some PTEs (e.g., As, Pb, Cd, and Hg) are highly toxic for plants even at low concentrations, while others (e.g., Cu, Ni, Mo, and Zn) are plant micronutrients, but can become phytotoxic at high concentrations (Adamo et al. 2014). Use of agrochemicals, fertilizers, and organic amendments, irrigation with wastewater, as well as illegal activities like waste burning or disposal are the primary anthropogenic causes of agricultural soil contamination by PTEs (Capra et al. 2014). The land application of organic amendments, such as compost, digestate, and stabilized sewage sludge, is an agronomical practice increasingly employed worldwide, which meets the rising necessity of recycling by-products and increasing the soil organic matter (OM) content (Sharma et al. 2017). However, if not carefully controlled and selected, these amendments can enrich the soil with PTEs up to the point of exceeding the toxicity thresholds (McGrath et al. 2001).

Accumulation of PTEs in agricultural soils does not always represent a real risk for crops, due to both a certain “soil buffer capacity”, which is based on the PTEs stabilization by OM and soil minerals (through sorption, complexation, and precipitation mechanisms), and to plants selectivity and exclusion mechanisms towards PTEs (Gattullo et al. 2017, 2020). In this regard, Brunetti et al. (2012) did not observe any dangerous accumulation of PTEs in straw and grain of durum wheat (*Triticum durum* Desf.) and barley (*Hordeum vulgare* L.) grown on an agricultural soil seriously polluted by Cr and other PTEs as a result of the soil amendment with tannery waste-derived materials. Further studies on the same site revealed that the presence of extremely high soil OM content enabled the immobilization of PTEs as well as preventing the oxidation of Cr, thus reducing the risks of their uptake by plants (Gattullo et al. 2020).

PTEs mobility and plant-availability are influenced by many factors, including PTEs’ total concentration and speciation in soil, as well as by soil physico-chemical characteristics, such as pH, OM content, nature, and amount of clay minerals and Al, Mn, and Fe (hydr)oxides, redox potential, etc. (Violante et al. 2010; Olaniran et al. 2013; Mahmoud and Ghoneim 2016). Among the soil properties affecting the PTEs mobility, the soil temperature has so far been poorly investigated. High temperatures, such as those occurring during a fire, can increase the rate of soil OM mineralization and mineral weathering thus causing the release of PTEs associated with these soil phases (Certini 2005; Abraham et al. 2017). Many agricultural areas worldwide are increasingly interested by fire occurrence due to the rising aridity, global warming, and land abandonment (Terzano et al. 2021). Fire can also be intentionally adopted for stubble burning, in order to prepare the soil for the subsequent cultivation cycle (Kumar et al. 2015). Additionally, burning of wastes illegally landfilled or disposed in agricultural soils is increasingly occurring, especially in degraded environments. Risks for food safety and human health need to be monitored when fires take place on agricultural soils polluted by PTEs.

Several studies revealed that high temperatures can favor the release of PTEs through the combustion of vegetation and OM, as well as from their sequestered phases in soil (Odigie and Flegal 2011, 2014; Kristensen et al. 2014; Burton et al. 2016; Odigie et al. 2016; Terzano et al. 2021). Fire can also alter the speciation of PTEs, enhancing in some cases the risks for both the environment and living organisms. For instance, a partial Cr oxidation can occur in Cr(III)-polluted soils after fire events, with formation of highly toxic and mobile Cr(VI) forms (Panichev et al. 2008; Burton et al. 2019). To our knowledge, there is a general lack of research on PTEs mobilization and speciation in agricultural soils after fires, and on the consequences for PTEs uptake by crops.

In the present study, the bioavailability of Cr and other PTEs (Zn, Pb, and Cu) and their uptake by durum wheat plants have been evaluated before and after laboratory simulated fires performed on a PTEs-polluted agricultural soil. Plant experiments were run using the RHIZOtest, a standardized biotest (ISO 16198:^2015^) largely adopted since 2000s and developed to assess the trace element availability to plants under rhizosphere conditions (Chaignon and Hinsinger 2003; Bravin et al. 2010; Pii et al. 2016; Puschenreiter et al. 2017). On the basis of changes of root exudation pattern and elemental distribution in plant organs, potential mechanisms of Cr uptake, translocation, and accumulation in plants are discussed.

## Materials and methods

### Site description and laboratory-simulated fires

Soil was sampled in a PTEs-polluted farmland situated in southern Italy (Altamura, Puglia region), typically cultivated with durum wheat. The site was previously characterized by Gattullo et al. (2020). The soil, classified as Calcaric Leptosol (IUSS Working Group WRB 2006), presented a high content of OM and Cr(III) as a result of the long-time amendment, performed until approximately 10–15 years ago, with tannery sludge-based biosolids. Besides Cr, Zn and Pb also exceeded the safety thresholds imposed by the Italian legislation (Italian Ministerial Decree n. 46/2019). An unpolluted control soil was also collected from an unamended nearby area.

Both polluted and unpolluted soils were air-dried, sieved at 2 mm, and then heated in a muffle furnace (LT 9/14/B180, Nabertherm GmbH, Lilienthal, Germany) up to 300 °C and 500 °C for 30 min. Soils (200 g for each sample) were heated in 20-cm diameter ceramic crucibles, forming a 0.7-cm-thick soil layer. The heating time and temperatures were selected on the basis of evidences reported by Li et al. (2012) for agricultural soils subjected to stubble burning. Three replicates were set up for each thermal treatment.

### Soil chemical characterization

Both heated and unheated soils were characterized for pH (Thomas 1996), electrical conductivity (EC) (Rhoades 1996), organic C content (Nelson and Sommers 1996), total N content (Bremner 1996), total and active CaCO_3_ (Loeppert and Suarez 1996), available P (Kuo 1996), cation exchange capacity (CEC), and exchangeable bases (Sumner and Miller 1996). The total content of PTEs was measured by energy-dispersive X-ray fluorescence (ED-XRF; NITON XL3t, Thermo Scientific Inc., Waltham, MA, USA), while the plant-available fraction (DTPA-extractable) of PTEs was determined according to Lindsay and Norvell (1978). Analysis of DTPA-extracts was performed by ICP-OES (Thermo iCAP 6000 series, Thermo Fisher Scientific Inc., Waltham, MA, USA). PTEs fractionation was assessed by means of a modified BCR sequential extraction procedure (Sahuquillo et al. 1999). Details on the analytical procedures adopted for ED-XRF and ICP-OES analyses, as well as for sequential extractions, are reported in Gattullo et al. (2020). Hexavalent Cr was extracted from soil samples through an alkaline digestion, according to USEPA method 3060A (USEPA 1996). Then, total Cr(VI) concentration in digests was determined by the colorimetric assay with diphenylcarbazide, according to USEPA method 7196A (USEPA 1992).

### RHIZOtest experiments

Seeds of durum wheat (Furio Camillo variety) were submerged overnight in water and then placed on filter paper moistened with water in the dark, for 3 d, to germinate. Seedlings were then transferred into cylindrical plastic pots (4 seeds for each pot) of 35-mm height and 34-mm diameter, capped on the bottom with a 30-µm mesh nylon membrane. Pots were subsequently inserted in a perforated floating platform (12 pots per platform), which was placed into a tank filled with 6 L of a germination solution (S1) containing 600 μM CaCl_2_ and 2 μM H_3_BO_3_ (Fig. S1a). Tanks were covered with an aluminum foil to prevent the light-induced inhibition during the seedling growth. After 4 d, S1 was replaced with a complete nutrient solution (S2) containing 0.5 mM KH_2_PO_4_, 2 mM KNO_3_, 2 mM Ca(NO_3_)_2_, 1 mM MgSO_4_, 0.2 μM CuCl_2_, 10 μM H_3_BO_3_, 2 μM MnCl_2_, 1 μM ZnSO_4_, 0.05 μM Na_2_MoO_4_, and 0.1 mM NaFe(III)EDTA (Fig. S1b). The S2 was continuously aerated by an air diffuser placed at the bottom of the tank, and renewed every 2 days. Experiments were performed in a climatic chamber, setting 14-h photoperiod, 25 °C day/20 °C night, 425 µmol m^−2^ s^−1^ light intensity, and 70% relative humidity. After 10 d of hydroponic cultivation, five pots with plants were harvested and processed (as described in the “Analysis of plants” section) to determine the root and shoot concentrations of PTEs and nutrients, and the root and shoot biomass at the end of the hydroponic pre-growth period (Fig. S1c). Conversely, the other 30 plant pots were used for the experiment with soil. Preliminarily, 150 g of each soil was incubated for 7 d in a plastic bag (drilled in the upper part to avoid anoxic conditions) and moistened with 50 mL of a weak macronutrient solution (S3) containing 50 μM KH_2_PO_4_, 2 mM KNO_3_, 2 mM Ca(NO_3_)_2_, and 1 mM MgSO_4_. The hydroponically grown plants were transferred on a 5-mm-thick soil layer, which was physically separated from the root mat by the nylon membrane, and maintained moist by a filter paper (placed under the soil layer) which was in contact with S3 (Fig. S1d). The S3 solution was inside a container (70 mm of height and 103 mm of diameter) placed below the plant pot (Fig. S1d). Five replicates for each soil sample were set up. After 7 d of soil–plant contact, plants were harvested, and after the root exudates collection, they were abundantly washed with deionized H_2_O, dried with a tissue paper and fresh-weighted. The setup of RHIZOtest experiments during the germination phase, the pre-growth hydroponics stage, and the soil–plant contact period was performed in accordance with the Rhizotest ISO standard procedure (ISO 16198:^2015^).

### Analysis of plants

Shoots and roots were separated using ceramic scissors, oven-dried at 60 °C for 3 d, weighted, and then pulverized using a vibro-milling system (MM 400, Retsch GmbH, Haan, Germany) (Allegretta et al. 2019). Shoot and root powders were then digested in HNO_3_ and H_2_O_2_, using a microwave digestion system (Multiwave GO, Anton Paar, Graz, Austria) (Gattullo et al. 2017). Total concentrations of P, S, K, Ca, Mn, Fe, Cu, Cr, Pb, and Zn were measured by means of total reflection X-ray fluorescence (TXRF) spectroscopy, using a S2 Picofox TXRF spectrometer (Bruker Nano GmbH, Berlin, Germany).

Some leaves were selected and immediately frozen with liquid nitrogen, keeping them flatted in a Petri dish, and then freeze dried for micro X-ray fluorescence (μ-XRF) analysis with a benchtop μ-XRF spectrometer (M4 Tornado, Bruker Nano GmbH, Berlin, Germany). The elemental distribution was measured on a rectangular area (3.8 × 5.0 mm) in the middle of the leaf. A line-scan acquisition was also performed to estimate the variation of element relative abundances across the leaf section.

Further details on TXRF and μ-XRF analyses are reported in Supplementary Information.

### Collection and analysis of root exudates

Root exudates were collected by immerging the whole root system into 20 mL deionized and aerated laboratory grade II water, for 4 h (Valentinuzzi et al. 2015). After the collection, plants were removed and the roots were dried with a paper towel and weighed. The exudate-containing solutions were frozen at − 20 °C and then freeze-dried. Immediately before analyses, the freeze-dried samples were resuspended in 3 mL 1:1 methanol:H_2_O and filtered with 0.45-µm syringe filters (Phenex-RC 0.45 µm—Phenomenex).

The content of total phenols in root exudates was determined with the Folin-Ciocalteu method (Atanassova et al. 2011), while the content of total flavonoids was determined following the Miliauskas et al. (2004) protocol. Total flavonols were quantified according to the Yermakov method (Mickelsen and Yamamoto 1958), and chelating compounds were determined using a modified protocol of the Chrome Azurol S (CAS) method (Shenker et al. 1995). Organic acids were determined by high-performance liquid chromatography (HPLC), as reported in Hullot et al. (2021).

### Statistical analysis

Data were initially tested for normality using the Shapiro–Wilk normality test. Data with a normal distribution were statistically analysed by one-way ANOVA and Tukey’s post hoc test using SigmaPlot 12.0 software (Systat Software Inc., Düsseldorf, Germany). Data of root exudates without a normal distribution were analyzed by the Kruskal–Wallis test with Dunn’s post hoc test (Ostertagová et al. 2014; Ott and Longnecker 2015), while *t*-test was performed in the case of two treatments comparison.

## Results and discussion

### Soil chemical characterization

Both heating treatments (at 300 °C and 500 °C) significantly altered (*p* ≤ 0.05) most of the soil chemical properties of either the polluted or unpolluted soil (Table 1). Soil pH significantly increased in both soils after heating at 500 °C, in agreement with the findings of Terefe et al. (2008). This increase might be due to degradation of organic acids and release of oxides, hydroxides, carbonates, and cations through ashes, as well as to the possible exchange of H^+^ with base cations on the exchange sites (Terefe et al. 2008; Terzano et al. 2021). The EC strongly increased in both soils after heating, to a greater extent at 300 °C than at 500 °C. Terefe et al. (2008) obtained similar results and attributed the increase of EC at 300 °C to the release of soluble inorganic ions from the exchange complexes and from OM combustion, while they ascribed the EC decline to the formation of base oxides and to their entrapment into coarser particle that were being formed at 500 °C. Soil combustion led to a significant decrease of CEC with increasing temperatures in both unpolluted and polluted soils, which might be related to the loss of negatively charged organic and inorganic soil colloids (Certini 2005). High temperatures also affected organic C and total N content, which decreased by approximately 80–90% in both soils after heating at 500 °C. These modifications were related to OM mineralization, which usually begins at temperatures between 130 °C and 200 °C with the degradation of lignin and hemicellulose, then becomes substantial at temperatures between 200 °C and 300 °C, and is almost completed at about 500 °C (Giovannini et al. 1990). A remarkable increase of available P was observed in the soils under investigation at increasing temperatures, which might be ascribed to the transformation of organic P into orthophosphate (Certini 2005). Total CaCO_3_ content varied only in the control soil, decreasing after heating, whereas active CaCO_3_ content changed only in the polluted soil, increasing by approximately 30% after heating at 500 °C. The concentration of exchangeable bases followed the sequence Ca^2+^ > K^+^ ≥ Mg^2+^ > Na^+^ in all the soils, except for the polluted soil heated at 500 °C, where the order was Ca^2+^ > Mg^2+^ > K^+^ > Na^+^. The exchangeable Ca^2+^ significantly decreased in heated soils as it probably precipitated as Ca-phosphate (Badía and Martí 2003). Conversely, exchangeable Na^+^ strongly increased of about threefold after heating both soils at 300 °C and 500 °C. No similar evidence was reported in the literature, except for a study of Guerrero et al. (2005).

**Table 1:** Chemical properties of unpolluted and polluted soils, before (unheated) and after fire simulations at 300 °C and 500 °C (mean ± standard deviation, *n* = 3). Within each soil (unpolluted or polluted), data with different letters in the row are significantly different according to one-way ANOVA and Tukey’s post hoc test (*p* < 0.05)

		Unpolluted			Polluted		
		Unheated	300 °C	500 °C	Unheated	300 °C	500 °C
pH (KCl)		7.3 ± 0.1^b^	7.1 ± 0.1^c^	7.6 ± 0.1^a^	7.1 ± 0.1^c^	7.7 ± 0.1^b^	8.2 ± 0.1^a^
EC (mS cm^−1^)		0.13 ± 0.01^c^	2.80 ± 0.20^a^	1.84 ± 0.08^b^	0.24 ± 0.03^c^	4.37 ± 0.49^a^	2.63 ± 0.74^b^
CEC (cmol_(+)_ kg^−1^)		50.8 ± 5.8^a^	36.6 ± 1.3^b^	19.8 ± 2.9^c^	76.2 ± 10.0^a^	42.1 ± 6.7^b^	32.6 ± 4.3^b^
Total CaCO_3_ (g kg^−1^)		165.7 ± 11.0^a^	93.2 ± 10.8^b^	82.5 ± 6.5^b^	202.4 ± 17.2 ns	234.4 ± 25.9 ns	240.4 ± 30.4 ns
Active CaCO_3_ (g kg^−1^)		13.6 ± 1.2^ab^	10.3 ± 3.1^b^	18.7 ± 1.3^a^	116.4 ± 15.8^b^	76.8 ± 1.9^c^	149.1 ± 4.4^a^
Organic C (g kg^−1^)		48.8 ± 6.5^a^	33.7 ± 3.3^b^	6.5 ± 0.8^c^	135.9 ± 20.2^a^	61.3 ± 6.9^b^	18.0 ± 3.2^c^
Total N (g kg^−1^)		3.9 ± 0.5^a^	3.4 ± 0.2^a^	1.0 ± 0.1^b^	15.0 ± 1.5^a^	10.9 ± 1.04^b^	2.5 ± 0.2^c^
Available P (mg kg^−1^)		4.6 ± 0.2^c^	95.4 ± 5.3^a^	48.4 ± 8.5^b^	182.8 ± 23.9^b^	184.9 ± 9.3^b^	397.1 ± 44.4^a^
Exchangeable bases(cmol_(+)_ kg^−1^)	Ca^2+^	28.4 ± 1.9^a^	19.3 ± 1.2^b^	11.8 ± 2.7^c^	48.3 ± 3.8^a^	30.2 ± 1.4^c^	38.9 ± 1.9^b^
	Mg^2+^	1.1 ± 0.1^ ns^	0.7 ± 0.1^ ns^	0.8 ± 0.1^ ns^	2.0 ± 0.3^ ns^	1.6 ± 0.6 ns	1.8 ± 0.5 ns
	Na^+^	0.10 ± 0.01^b^	0.36 ± 0.03^a^	0.31 ± 0.15^a^	0.10 ± 0.02^c^	0.41 ± 0.01^a^	0.27 ± 0.07^b^
	K^+^	1.9 ± 0.41^ ns^	1.7 ± 0.3^ ns^	1.1 ± 0.3^ ns^	2.2 ± 0.5^a^	1.3 ± 0.3^ab^	0.8 ± 0.1^b^

*ns* not significant (*p* > 0.05)

Alteration of the soil chemical properties caused by fire events can indirectly affect PTEs behavior (Terzano et al. 2021). The most concentrated PTEs in the polluted soil were, in the order, Cr, Zn, Cu, and Pb (Table 2); thus, their distribution and mobility in soil were investigated. After the thermal treatment at 500 °C, PTEs’ total concentration in the polluted soil significantly increased by a minimum of 11% for Cr to a maximum of 55% for Pb (Table 2). This soil enrichment by PTEs was only apparent, being mainly ascribable to OM mineralization.

**Table 2:** Total and available (DTPA-extractable) concentrations of potentially toxic elements and Cr(VI) total content in unpolluted and polluted soils, before (unheated) and after fire simulations at 300 °C and 500 °C (mean ± standard deviation, *n* = 3). Within each soil (unpolluted or polluted), data with different letters in the row are significantly different according to one-way ANOVA and Tukey’s post hoc test (*p* < 0.05)

			Unpolluted			Polluted		
			Unheated	300 °C	500 °C	Unheated	300 °C	500 °C
Cr	Total	mg kg ^−1^ DW	65 ± 11^ ns^	60 ± 9^ ns^	46 ± 6^ ns^	5160 ± 35^c^	5397 ± 80^b^	5715 ± 13^a^
	Available		0.01 ± 0.003^b^	0.15 ± 0.04^a^	0.14 ± 0.05^a^	0.32 ± 0.04^c^	26 ± 4^b^	105 ± 9^a^
Cr(VI)	Total		b.d.l.	b.d.l.	b.d.l.	b.d.l.	27 ± 7^b^	152 ± 44^a^
Zn	Total		69 ± 3^b^	70 ± 1^b^	82 ± 1^a^	1270 ± 10^c^	1618 ± 19^b^	1835 ± 23^a^
	Available		1.6 ± 0.3^b^	3.2 ± 0.7^a^	2.4 ± 0.5^ab^	208 ± 24^a^	136 ± 18^b^	59 ± 7^c^
Cu	Total		32 ± 4^b^	35 ± 3^ab^	41 ± 2^a^	134 ± 5^c^	175 ± 13^b^	201 ± 11^a^
	Available		1.5 ± 0.2^b^	2.5 ± 0.5^a^	2.0 ± 0.1^ab^	14 ± 3^a^	1.6 ± 0.6^c^	8.8 ± 0.3^b^
Pb	Total		10 ± 3^ ns^	9 ± 1^ ns^	13 ± 3^ ns^	114 ± 3^c^	148 ± 3^b^	177 ± 2^a^
	Available		1.0 ± 0.4^ ns^	1.9 ± 0.5^ ns^	1.4 ± 0.6^ ns^	5.1 ± 0.7^b^	5 ± 1^b^	8.3 ± 0.5^a^

*DW* dry weight; *b.d.l.* below detection limit; *ns* not significant (*p* > 0.05)

**Fig. 1:**
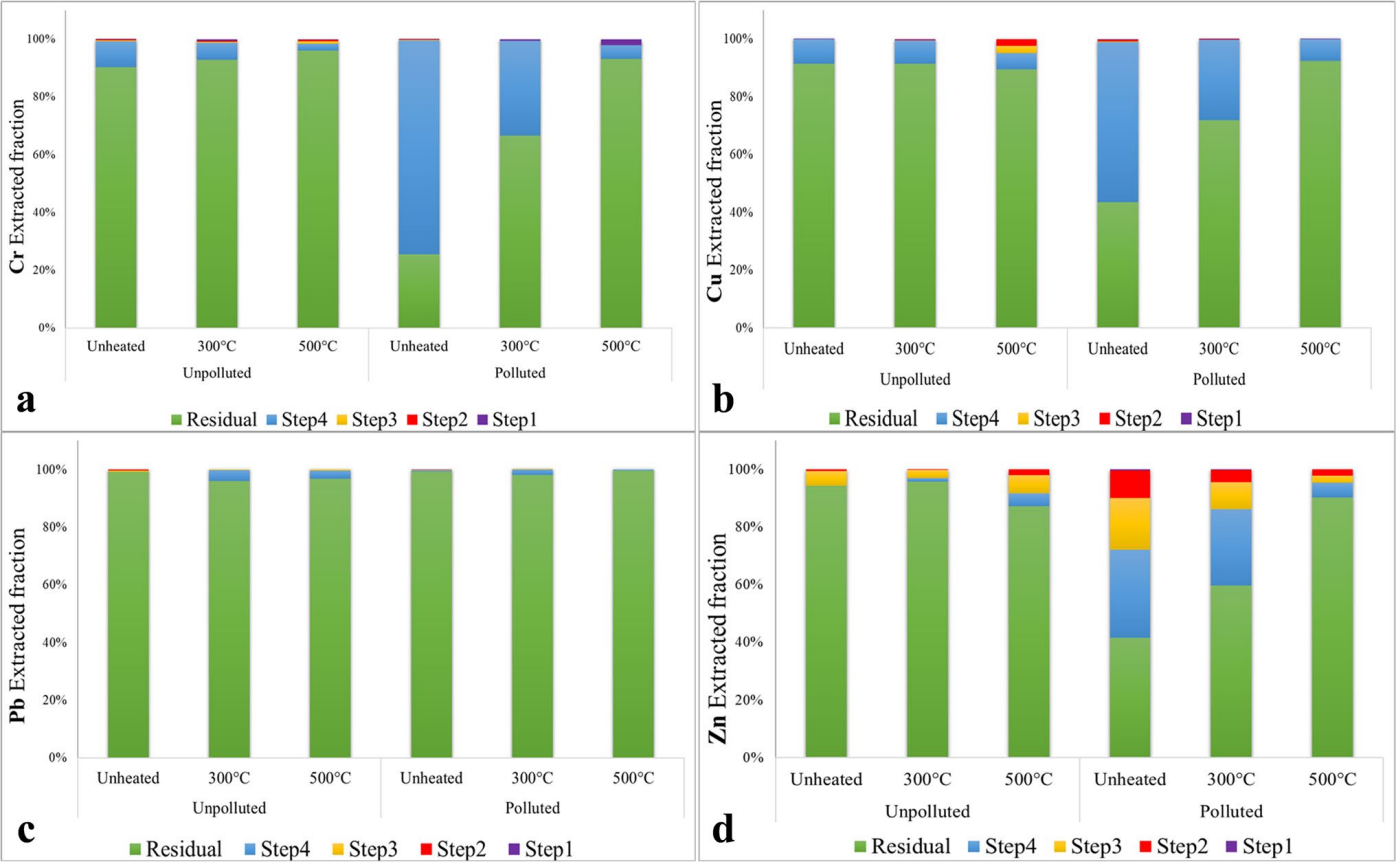
PTEs’ fractionation of (**a**) Cr, (**b**) Cu, (**c**) Pb, and (**d**) Zn, in both unpolluted and polluted soils before (unheated) and after fire simulations at 300 °C and 500 °C. The four steps of BCR sequential extraction procedure correspond to the exchangeable (Step 1), acid-soluble (Step 2), reducible (Step 3), and oxidizable (Step 4) fractions. “Residual” corresponds to the fraction bound to soil recalcitrant phases remaining at the end of Step 4

The PTEs fractionation in soils before and after the heating treatments is reported in Fig. 1. The distribution of Cr and Cu in the polluted soil significantly changed with increasing temperature, specifically their amount in the oxidizable fraction decreased (Step 4 of sequential extraction procedure), whereas their amount in the soil residual fraction increased (Fig. 1a, b). Moreover, in the polluted soil, the amount of Zn bound to the soil residual fraction increased with increasing temperature, at the expenses of fractions associated with carbonates (Step 2 of sequential extraction procedure), reducible compounds (Step 3), and oxidizable compounds (Step 4) (Fig. 1d). Analogously, Memoli et al. (2020) observed that the fraction of Cr, Pb, and Cu bound to soil OM and/or to sulfides (Step 4 of sequential extraction procedure) significantly decreased after a wildfire, whereas the fraction of these PTEs in the soil residual fraction increased. As the OM content decreased with increasing heating temperatures, its primary role in metal-binding decreased as well, thus favouring PTEs’ release and redistribution in other soil fractions. The Cr soluble and exchangeable fraction (Step 1 of sequential extraction procedure) significantly increased in the polluted soil at increasing heating temperatures (Fig. 1a). Specifically, it raised from 0.5 mg kg^−1^ in the unheated soil to 36 and 124 mg kg^−1^ in soil heated at 300 °C and 500 °C, respectively. This mobile Cr fraction might include both labile Cr(III) forms and soluble Cr(VI) forms. Approximately 99% Pb was immobilized in the soil residual fraction, regardless of the thermal treatments (Fig. 1c), thus revealing very limited environmental risks. No relevant changes in PTEs distribution were observed in the control soil after fire simulation (Fig. 1). Results of the PTEs’ plant-available fraction (assessed through DTPA-extractions) are reported in Table 2. In the polluted soil, the Pb DTPA-extractable fraction slightly increased only after the treatment at 500 °C. Conversely, the Cu available fraction in the polluted soil decreased by approximately 90% and 40% after soil heating at 300 °C and 500 °C, respectively. Similarly, Sitlhou and Singh (2014) found that soil heating reduced the Cu DTPA-extractable content in the top layer (0–5 cm). The Zn DTPA-extractable fraction also decreased by 35% and 72% after soil heating at 300 °C and 500 °C, respectively. On the contrary, García-Marco and Gonzalez-Prieto (2008) observed a significant increase of the Zn DTPA-extractable fraction 1 and 90 days after soil heating. Despite the very high Cr concentration in the polluted soil, the Cr DTPA-extractable fraction was extremely low before soil heating, accounting to only 0.3 mg kg^−1^ (Table 2). In fact, Cr was present in soil as immobile Cr(III) forms or bound to OM, and consequently, it was scarcely bioavailable for plants (Gattullo et al. 2020). After soil heating at 300 °C and 500 °C, Cr-DTPA extractable fraction significantly increased reaching values up to 26 and 106 mg kg^−1^, respectively. The heating treatments also caused a partial oxidation of Cr(III) to Cr(VI) (Table 2). It is worth of notice that the Cr-available concentrations determined after heating at both 300 °C and 500 °C correspond almost exactly to the concentration of Cr(VI) determined in the heated soil and, in turn, also to the mobile fraction assessed by Step 1 of sequential extraction procedure. Therefore, it is reasonable to think that the partial oxidation of Cr(III) to Cr(VI) made Cr more mobile and potentially more bioavailable. In this case, DTPA had no role in Cr mobilization, since it can chelate cations but not anions, as in the case of Cr(VI) forms. The latter are simply soluble in aqueous solutions, and this explains the reason for the similarity between results obtained from Step 1 of sequential extraction procedure and results of DTPA extraction.

Cr(VI) was not detected in the control soil, neither before nor after the thermal treatments, nor in the unheated polluted soil (detection limit for Cr(VI): 0.2 mg kg^−1^). Conversely, Cr(VI) concentrations significantly above the Italian safety threshold established for agricultural sites (2 mg kg^−1^; Italian Ministerial Decree n. 46/2019) were detected after heating the polluted soil, thus underlying the potential risk for environmental and human health after fire events on PTE-polluted areas.

### Root/shoot biomass ratio

The root/shoot biomass ratio (R/S) of plants after 7 d of contact with the two soils (unpolluted and polluted), unheated or heated, is reported in Fig. S2. Changes of R/S can be indicative of biotic and abiotic stresses, which may influence the reallocation of plant metabolites between the above-ground and below-ground biomass. Plants preferentially allocate more biomass to the roots if their growth is limited by belowground factors (e.g., nutrient deficiency, salinity, drought), in order to enhance the uptake of nutrients and water (Franco et al. 2011; Yang et al. 2018). In this study, plants showed similar R/S ratio regardless of the soil type and heating treatment. Most likely, the period of contact between plants and soil was not long enough to appreciate relevant modifications of this biometric parameter. Indeed, the RHIZOtest system is designed to investigate the rhizosphere processes underpinning the transfer of nutrients and PTEs from soil to plant, but not the plant growth, due to the limited time of contact between plant and soil (Bravin et al. 2010).

### Accumulation of PTEs and nutrients in roots and shoots

**Fig. 2:**
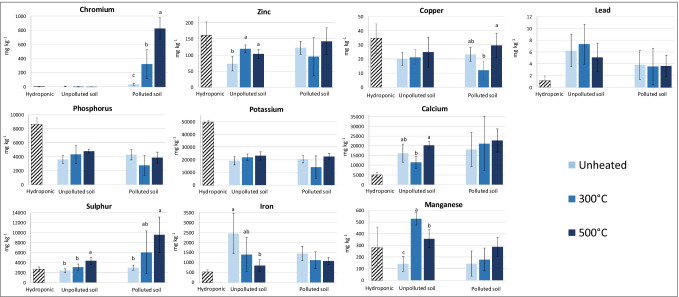
Concentrations (mg kg^−1^ dry weight) of PTEs and essential elements in roots of durum wheat after 7d of contact with the polluted or unpolluted soil, unheated or heated at 300 °C and 500 °C. The concentrations measured in roots of plants harvested at the end of the pre-growth hydroponic period are also reported for comparison (Hydroponic). The vertical line on each bar indicates the standard deviation (*n* = 5). Different letters indicate significant differences within plants grown on the same type of soil (unpolluted or polluted), according to one-way ANOVA and Tukey’s post hoc test (*p* < 0.05)

**Fig. 3:**
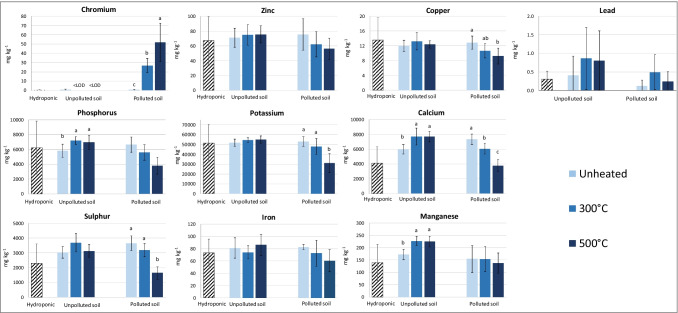
Concentrations (mg kg^−1^ dry weight) of PTEs and essential elements in shoots of durum wheat after 7d of contact with the polluted or unpolluted soil, unheated or heated at 300 °C and 500 °C. The concentrations measured in shoots of plants harvested at the end of the pre-growth hydroponic period are also reported for comparison (hydroponic). The vertical line on each bar indicates the standard deviation (*n* = 5). Different letters indicate significant differences within plants grown on the same type of soil (unpolluted or polluted), according to one-way ANOVA and Tukey’s post hoc test (*p* < 0.05)

The concentrations of PTEs and essential elements, expressed on dry weight basis, were measured in roots (Fig. 2) and shoots (Fig. 3) of durum wheat after 7 d of contact with the polluted or unpolluted soil (heated or unheated).

The main and most striking difference between plants grown on the polluted soils and plants grown on the unpolluted soils was in the uptake and translocation of Cr. A Cr concentration of 3 mg kg^−1^ was measured in roots at the end of the pre-growth hydroponic period (pre-growth concentration). Only 7.5 mg kg^−1^ of Cr was detected in roots of plants grown on the unpolluted soil, regardless of the soil heating treatment (Fig. 2). Transfer of Cr from the unpolluted soils to plants was negligible due to both the low concentration of Cr in these soils (Table 2) and to its immobilization as Cr(III) in the most recalcitrant soil fractions (Fig. 1). Conversely, plants grown in the polluted soil accumulated Cr in roots at a greater extent, with a significantly increasing trend as the treatment temperatures increased (Fig. 2). The Cr accumulation in roots of plants grown in the unheated polluted soil was not negligible (37 mg kg^−1^), notwithstanding the very low Cr mobility in soil. Most likely, the root exudation of chelating compounds (Table 3) mobilized Cr(III) from soil, thus allowing plants to acquire Cr. The effect of soil heating on the transfer of Cr from the polluted soil to plants was remarkable. In fact, Cr concentration in roots of plants grown in 300 °C- and 500 °C-heated soil raised up to 325 and 829 mg kg^−1^, respectively (Fig. 2). This trend was consistent with the increase, at increasing soil heating temperatures, of DTPA-extracted Cr, mobile fraction of Cr as assessed by step 1 of sequential extraction procedure, as well as with the partial oxidation of Cr(III) to the more soluble and bioavailable Cr(VI) form (Table 2). Similarly, although to a lesser extent, Hafez et al. (1979) measured from 1.5 to 3 times higher concentrations of Cr in roots of maize (*Zea mays* L.) grown on a soil heated at 300 °C, compared to the plants grown on the unheated soil. Chromium translocation to shoots followed the same trend observed for roots (Fig. 3). Chromium concentrations of 27 and 52 mg kg^−1^ were measured in the shoots of plants grown in the polluted soil heated at 300 °C and 500 °C, respectively, whereas values below or close to the detection limit (5 µg kg^−1^) were found in the other treatments, including plants at the end of the pre-growth hydroponic period. Background leaf concentrations below 1 mg kg^−1^ are reported in the literature for a wide range of soil Cr concentrations (Smith et al. 1989).

**Table 3:** Root exudates of durum wheat plants grown on unpolluted or polluted soils before (unheated) and after fire simulations at 300 °C and 500 °C. Within each soil type (unpolluted or polluted), data (mean ± standard deviation, *n* = 5) with different letters in the row are significantly different according to one-way ANOVA and Tukey’s post hoc test (*p* < 0.05)

		Unpolluted			Polluted		
		Unheated	300 °C	500 °C	Unheated	300 °C	500 °C
Total phenols (µmol gallic acid equivalent h^−1^ g^−1^ DW)		87 ± 41^b^	248 ± 82^a^	91 ± 42^b^	119 ± 69^ ns^	207 ± 244^ ns^	75 ± 39^ ns^
Total flavonols (nmol rutin hydrate equivalent h^−^1 g^−1^ DW)		b.d.l.	39 ± 10^a^	24 ± 8^b^	17 ± 7^ ns^	21 ± 6^ ns^	22 ± 6^ ns^
Total flavonoids (nmol rutin hydrate equivalent h^−1^ g^−1^ DW)		b.d.l.	35 ± 33^ ns^	98 ± 63^ ns^	31 ± 24^ ns^	22 ± 15^ ns^	b.d.l
Chelating compounds **(**µmol EDTA equivalent h^−1^ g^−1^ DW)		b.d.l.	2.1 ± 1.9^ ns^	0.7 ± 1.1^ ns^	1.2 ± 1.2^ ns^	0.6 ± 0.2^ ns^	b.d.l.
Total acidity (µmol organic acids h^−1^ g^−1^ DW)		0.1 ± 0.1^ ns^	b.d.l.	b.d.l.	0.2 ± 0.1^ ns^	b.d.l.	b.d.l.

*b.d.l.* below detection limit; *DW* dry weight; *ns* not significant (*p* > 0.05)

The Zn concentrations measured in roots and shoots of plants grown in the polluted soil at the three temperatures were similar to the pre-growth concentrations. Therefore, despite of the high values of DTPA-extracted Zn in these soils (Table 2), no net plant uptake occurred for this element. As reported by Marschner (1986) and Hart et al. (1998), Zn uptake can be inhibited by high concentrations of other divalent cations, mainly Ca^2+^. Indeed, high concentrations of exchangeable Ca^2+^ were detected in the polluted soil, even after soil heating (Table 1), and higher levels of Ca were measured in the roots and shoots of plants grown on these soils compared to hydroponically grown plants (Figs. 2, 3). All these pieces of evidence strengthen the hypothesis that Zn uptake was inhibited by Ca. Plants grown in the unpolluted soil also showed root and shoot Zn concentrations comparable or even lower than the pre-growth concentrations, although heating slightly enhanced the accumulation of this PTE in durum wheat roots. These results are in agreement with the very low values of DTPA-extractable Zn in the unpolluted soil, at the different temperatures of treatment (Table 2). Likewise, Nishita et al. (1970) observed that heating uncontaminated soils at temperatures up to 500–600 °C does not influence the Zn leaf content of bean plants (*Phaseolus vulgaris* L.).

No net Cu accumulation was evidenced both in roots and shoots of plants after the contact with soil, regardless of the soil type and heating treatment (Figs. 2, 3). This result was in agreement with the low Cu concentration in the unpolluted soil and the low mobility of this element in both polluted and unpolluted soils. Accumulation of Cu in roots of plants grown in the polluted soil was significantly affected by the temperature of treatment (Fig. 2) and followed a trend similar to that of the DTPA-extractable Cu in soil (Table 2). Plants grown in the polluted soil also showed a significantly decreasing translocation of Cu in shoots as the temperature of soil heating increased (Fig. 3). No effect of soil heating was recorded for Cu accumulation in roots and shoots of plants grown in the unpolluted soil. Conversely, Nishita et al. (1970) observed a proportional increase of Cu leaf concentrations as the soil heating temperature increased.

The pre-growth concentration of Pb in roots was very low (1.2 ± 0.8 mg kg^−1^), as expected. Plants grown in contact with the polluted soil contained approximately 3.7 mg kg^−1^ of Pb in roots, regardless of the soil heating treatment. Transfer of this metal from the polluted soil to plants was negligible since more than 98% of Pb was bound to the soil residual fraction (Fig. 1c), and the DTPA-extractable fraction did not exceed few mg kg^-1^ (Table 2). A higher root accumulation of Pb was recorded in plants grown in the unpolluted soil, reaching a maximum value of 7.4 mg kg^−1^ in the 300 °C-heated soil. Nonetheless, these values were close to the background concentrations reported in the literature for wheat plants (Chandra et al. 2009). Very little Pb was translocated to shoots, in agreement with findings reported in the literature (Steinness 2013). Soil heating did not significantly affect the Pb accumulation in both roots and shoots.

Concentrations of P and K in plant roots were similar for both soils and for the three temperatures, and were lower than the pre-growth concentration (Fig. 2). This finding appeared in contrast with the high values of available P in soils (Table 1) but, as demonstrated by Sánchez-Alcalá et al. (2015), Olsen P can be overestimated in calcareous soils. A fraction of available P might have precipitated as Ca-phosphate, becoming unavailable for plant uptake. Unlike P and K, a strong net accumulation of Ca was recorded in roots for each treatment, in accordance with the high values of exchangeable Ca^2+^ and active CaCO_3_ found in these soils (Table 1). No relation between the soil thermal treatment and Ca uptake was observed in plants grown in the polluted soil. The root concentrations of S significantly increased with the increase of the soil heating temperature, for both soils (Fig. 2). A net uptake of this nutrient was recorded in plants grown in the 500 °C-heated polluted soil. The Fe concentrations in roots decreased with increasing soil-heating temperatures (Fig. 2). Nevertheless, the reduction was significant only for the unpolluted soil and was possibly ascribable to the increased acquisition of Mn, which can compete with Fe and impair its uptake. Manganese is among the elements that most accumulate in soil after a fire, deriving mainly from ashes of the combusted vegetation (Campos et al. 2016; Terzano et al. 2021), and some studies report that Mn concentrations in plants increase after soil heating (Nishita et al. 1970; Kang and Sajjapongse 1980). Manganese concentrations in roots of plants grown in the polluted soil also increased with the increase of soil-heating temperature, although non-significantly.

Plants grown in the polluted soil showed a reduced translocation of S, K, Ca, Cu, and, although non-significantly, of P, Fe, and Zn with the increase of soil-heating temperature, conversely Cr translocation increased (Fig. 3). Soil heating differently affected the element translocation in plants grown in the control soil. For this group of plants, translocation increased with the soil-heating temperature for P, Ca, and Mn, while no significant variation was observed for S, K, Cr, Fe, Cu, and Zn. Based on these pieces of evidence, it can be hypothesized that Cr translocation in plants grown in polluted soils subjected to fire strongly impaired translocation of macro and micronutrients in plant shoots. Similarly, Barceló et al. (1985) observed a reduction of translocation of P, K, Zn, Cu, and Fe in bean plants exposed to Cr.

### Potential mechanisms of Cr uptake, translocation, and accumulation in plants

Risk of PTEs transfer from the polluted soil to durum wheat plants after soil fire was observed only for Cr. In order to unravel the potential mechanisms involved in the acquisition and translocation of this element by plants, the root exudation pattern as well as the Cr distribution inside plant and its associations with other elements were investigated.

Chromium is a non-essential element for plants and, especially in the hexavalent form, it can be highly toxic, impairing plant growth and development (Shanker et al. 2005). Plants do not possess specific carriers nor channels for Cr uptake but, especially when growing in Cr-rich substrates, they can absorb it through the transport proteins typically involved in the acquisition of mineral nutrients (Shanker et al. 2005; Ao et al. 2022). As widely recognized, Cr can be taken up both as Cr(III), through a passive mechanism, and as Cr(VI), exploiting the carrier system of sulfate or phosphate, due to the chemical structure similarity of these anions (Skeffington et al. 1976; Smith et al. 1989; Zayed et al. 1998; Hamilton et al. 2020; Ao et al. 2022).

Plants grown in the unheated polluted soil exuded different classes of metabolites (e.g., phenols, flavonoids, flavonols, organic acids). In contrast, only phenols and organic acids were detected in root exudates of plants grown on unheated control soil (Table 3). Root exudates are known to be determinant for plant performance (Canarini et al. 2019; Vives-Peris et al. 2019), especially for the nutrient uptake (Chen et al. 2017; Mimmo et al. 2018), interaction with rhizospheric microorganisms (Haichar et al. 2014) as well as for heavy metal detoxification (Zeng et al. 2008; Fan et al. 2016; Ghori et al. 2019). Most likely, durum wheat plants tried to react to PTEs contamination enhancing and diversifying the root exudation pattern. When plants grown in the unheated polluted soil were compared with plants grown in the heated polluted soils, exudation of phenolic compounds generally did not change, except for total flavonoids, which were not released at all in the treatment at 500 °C (Table 3). Conversely, exudation of organic acids and chelating compounds decreased with the increasing temperature, and none of the two compound classes was detected in exudates collected from plants grown in the 500 °C-heated polluted soil (Table 3), notwithstanding the high Cr uptake found in these treatments (Figs. 2, 3). Based on these pieces of evidence, it can be asserted that chelating compounds and organic acids were most likely responsible for Cr uptake in the unheated soil, where all Cr was bound to OM and soil recalcitrant mineral fractions as Cr(III). It is known that organic acids can mobilize Cr(III) by forming organically bound Cr(III)-complexes which can be taken up by plants, including wheat (Srivastava et al. 1999a,b). Phytosiderophores, belonging to the chelating compounds, might have also been involved in the uptake of Cr(III), as proved by Liu et al. (2011).

Conversely, Cr mobilization in the heated polluted soils, where both Cr(III) and Cr(VI) forms were present, seemed not to be related to the exudation of chelating compounds and organic acids. Most likely, Cr uptake in the heated soils shifted more to Cr(VI) due to its higher mobility in soil and higher transmembrane transport efficiency (Ao et al. 2022) and, consequently, involved different routes of entry, such as sulfate or phosphate carriers. Looking at the uptake of Cr, P, and S by durum wheat plants in Cr-polluted soils, a similar trend was observed for Cr and S, whose concentrations in roots significantly increased at increasing soil heating temperatures, whereas no relation was observed between Cr and P uptake (Fig. 2). The higher accumulation of sulfate in conjunction with the higher accumulation of Cr(VI) has been found also in previous studies and seems to be related to the sulfate-induced metal tolerance rather than to the sharing of the same carriers (Lindblom et al. 2006; de Oliveira et al. 2016). Indeed, a competitive effect is typically observed between Cr uptake and S uptake when the two elements share the same transporters (Schiavon et al. 2008). Further investigations are required to clearly identify the transporters involved in the uptake of Cr by durum wheat plants, and routes other than the ones based on sulfate transporters can be also hypothesized.

Chromium was mainly accumulated in roots. As far as plants grown in the unheated polluted soil are concerned, the Cr root concentration was 74 times higher than the Cr shoot concentration, while in plants grown in the polluted soil heated at 300 °C and 500 °C the Cr root concentration was 12 and 16 times higher than the Cr shoot concentration, respectively. Similarly, Cr root concentrations from 10 to 100 times higher than in shoots are reported in the literature (Cervantes et al. 2001; Shanker et al. 2005; Ao et al. 2022). The reason for Cr accumulation in roots is attributable to its sequestration in root cortex cells, both at the cell wall level as Cr(III) and in vacuoles, as Cr(III) or Cr(VI) (Shanker et al. 2005). Chromium sequestration is a detoxification strategy adopted by plants along with another defence mechanism based on reduction of Cr(VI) to Cr(III) (Hamilton et al. 2020). Chromium reduction mainly occurs in the root cortex cells immediately after Cr intake (Zayed et al. 1998; Shanker et al. 2005), but also during Cr apoplastic transport to xylem vessels (Ao et al. 2022), and in aerial plant tissues (Cervantes et al. 2001). Chromium reduction in plants usually involves sulfur-containing compounds, such as cysteine, glutathione, sulfite, and thiosulfates (Whitacre 2010; Sinha et al. 2018). In the present study, a significant reduction of S concentration was observed in shoots of plants accumulating most Cr (i.e., plants grown in the polluted soil heated at 500 °C). This reduced translocation was possibly due to the enhanced synthesis of thiols in roots, in order to cope with Cr toxicity.

Once reduced, Cr became scarcely mobile and tended to be mainly retained in roots. Nevertheless, a fraction of Cr was translocated to shoots. Translocations through xylem are documented in several review papers (Shanker et al. 2005; Sinha et al. 2018; Ao et al. 2022), although a few studies focus on Cr speciation inside plant (Zayed et al. 1998; Hamilton et al. 2020; Park 2020). A fraction of Cr(III) can be loaded into xylem vessels, complexed by xylem sap ligands (mainly carboxylates) and acropetally transported (Juneja and Prakash 2005; Ao et al. 2022). Even Cr(VI) can be transported through xylem when the amount of Cr(VI) taken up exceeds the reducing capacity of root cells (Barceló and Poschenrieder 1997).

**Fig. 4:**
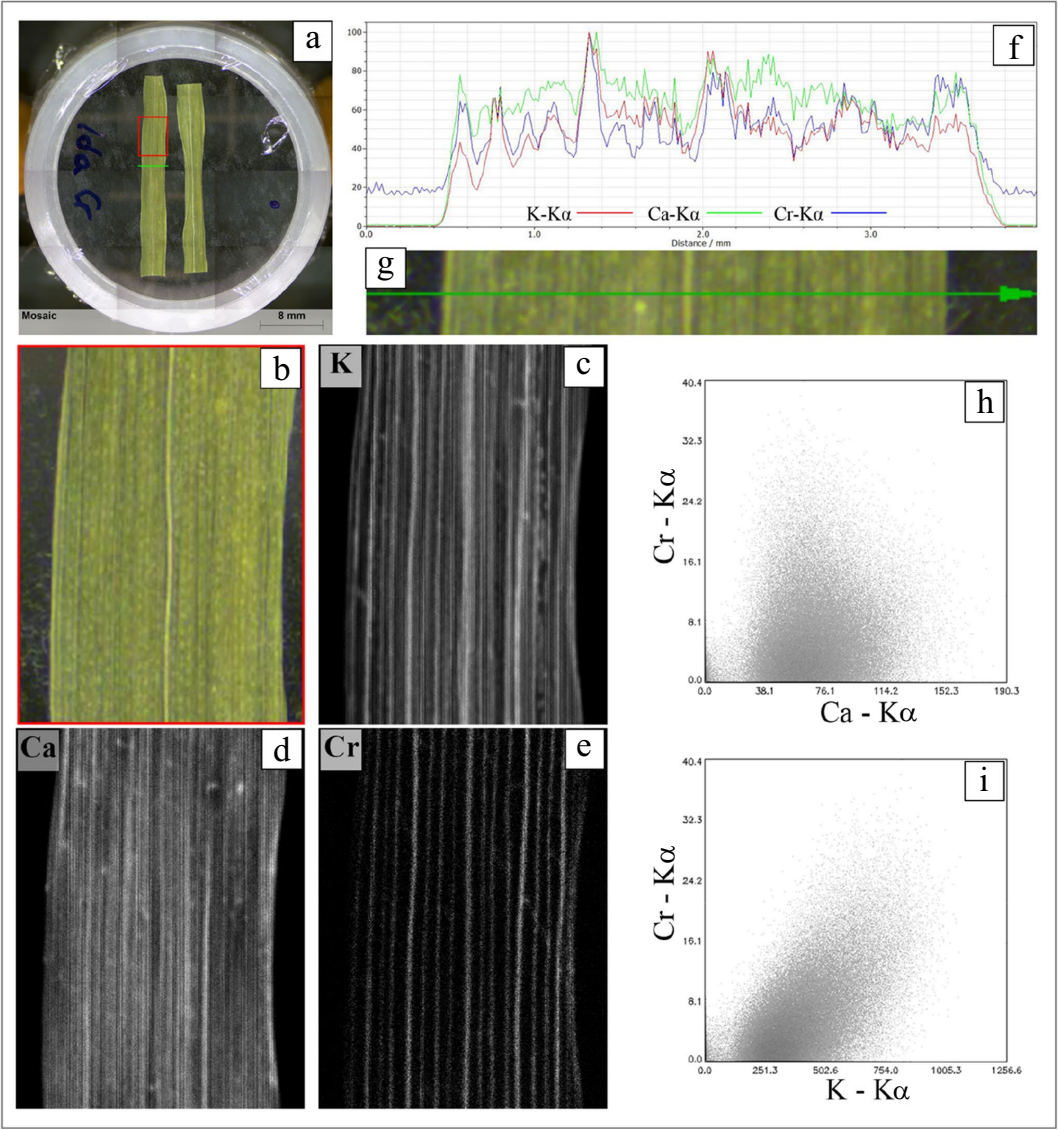
(**a**) Image of a durum wheat leaf analyzed by μ-XRF. The red rectangle corresponds to the area where μ-XRF maps were collected, while the green line is where the μ-XRF linescan was performed. (**b**) Enlarged image of the red rectangle in (**a**) where μ-XRF maps were collected. (**c**, **d**, **e**) μ-XRF distribution maps of K, Ca, and Cr, respectively. Brighter pixels in (**c**, **d**, **e**) correspond to relatively higher element concentrations. (**f**) Relative abundance of K, Ca, and Cr along the linescan. (**g**) Enlarged image of the area where the μ-XRF linescan (green line) was performed. (**h**) Scatterplot of Cr vs Ca and (**i**) Cr vs K fluorescence intensities

Distribution of Cr and its association with other elements was assessed by µ-XRF in leaves sampled from plants grown in the polluted soil heated at 500 °C (Fig. 4a). The acquisition of elemental maps in the middle part of the leaf (evidenced with the red rectangle in Fig. 4b) revealed that Cr was mainly distributed along the leaf veins (Fig. 4e). Because of the X-ray penetration inside the whole leaf cross-section, it cannot be claimed if Cr was distributed inside the xylem or in the phloem vessels, even if this latter possibility is less probable. Looking at the other elemental maps, K showed a very similar distribution compared to that of Cr (Fig. 4c), being principally distributed along the leaf veins, although its signal was more intense than that of Cr. The map of Ca revealed that this macronutrient was present both along the leaf veins and in the inter-vein spaces (Fig. 4d). Indeed, Ca is a structural element and is ubiquitous in leaf tissues. The line-scan acquisition done along the green transversal segment, traced in Fig. 4g, evidenced that the Cr signal overlapped almost perfectly that of K, while this correspondence was not so clear with Ca (Fig. 4f). The scatterplots of Cr-K and Cr-Ca signals obtained from the µ-XRF maps (Fig. 4h,i) confirmed that Cr was more correlated to K than to Ca. Calcium is a structural element, mainly concentrated in the cell walls and scarcely mobile, whereas K^+^ is the most abundant cation in the cytosol and is characterized by a high mobility, both at the cellular level and in the long-distance transport through xylem and phloem (Hawkesford et al. 2012). Based on the Cr distribution along leaf veins and on its correlation with K, we can hypothesize that Cr mainly occurred as mobile forms in durum wheat leaves, while only a minor fraction was retained by cell walls.

## Conclusions

RHIZOtest experiments using durum wheat plants allowed to assess the PTEs bioavailability in agricultural polluted soils treated at different temperatures to simulate fire events. The results obtained showed no evident risk of accumulation and translocation of Zn, Pb, and Cu in plants after fire simulations, whereas a high accumulation in roots and a significant translocation to shoots were observed for Cr. Despite this PTE was initially (before heating) very stably immobilized in the polluted soil, its partial release by the most recalcitrant soil phases and its partial oxidation to Cr(VI) induced by soil heating might have favored the Cr uptake by plants. The Cr form taken up by plants was most likely the fraction oxidized to Cr(VI), as also soil chemical analyses and root exudation patterns suggested. Indeed, exudation of cation complexing molecules was reduced at higher temperatures, but nonetheless a higher Cr accumulation in plant tissues was observed. The concomitant increase of Cr and S content in roots at increasing soil-heating temperatures was possibly related to S involvement in enhancing plant tolerance to Cr, as reported in previous studies. Once taken up, Cr mainly remained in roots, but part of it was translocated to shoots where it was likely present in mobile forms either inside leaf cells or in the xylem, showing a distribution similar to that of K.

This research shows how the high temperatures occurring during fire events can change PTEs fractionation and speciation in soil and cause the formation of more bioavailable PTEs chemical forms that, in turn, can be more easily taken up by plant roots and transferred to aerial parts. Such events can transform apparently safe environments into potentially dangerous sources of pollution that can then ultimately affect human health through food chain transfer or migration in surface water and groundwater.

## Supplementary Information

Below is the link to the electronic supplementary material.

Supplementary file1 (13.3 MB)

## Data Availability

Not applicable.
