# Supplementary material for: Fire-induced effects on the bioavailability of potentially toxic elements in a polluted agricultural soil: implications for Cr uptake by durum wheat plants
Source: Environ Sci Pollut Res Int. 2022 Aug 23;30(3):6358–72. doi: 10.1007/s11356-022-22471-5 (PMC9895035; doi:10.1007/s11356-022-22471-5)
Supplement: Supplementary file 1 — Supplementary file1 (13.3 MB) [file 11356_2022_22471_MOESM1_ESM.docx]

**Fire-induced effects on the bioavailability of potentially toxic elements in a polluted agricultural soil: Implications for Cr uptake by durum wheat plants**

Ida Rascio^a^, Concetta Eliana Gattullo^a*^, Carlo Porfido^a^, Ignazio Allegretta^a^, Matteo Spagnuolo^a^, Raphael Tiziani^b^, Silvia Celletti^b^, Stefano Cesco^b^, Tanja Mimmo^b^, Roberto Terzano^a^

^a^Department of Soil, Plant and Food Sciences, University of Bari “Aldo Moro”, Via G. Amendola n. 165/A - 70126 Bari, Italy

^b^Faculty of Science and Technology, Free University of Bozen-Bolzano, Piazza Università n. 5 -39100 Bolzano, Italy

**^*^Corresponding author:**

Dr. Concetta Eliana Gattullo

concettaeliana.gattullo@uniba.it

**1. Details on TXRF and μ-XRF analyses**

Total reflection X-ray fluorescence (TXRF) spectroscopy was used to measure the total concentrations of PTEs and nutrients in digested samples of plant roots and shoots. For this purpose, 10 µL of Ga standard solution (Sigma-Aldrich) were added to an aliquot of 1 mL of the digested sample as internal standard (final Ga concentration 10 mg L^-1^). Then, 10 µL of sample solution were deposited on a siliconized quartz reflector, dried at 50°C on a heating plate under a laminar flow hood, and finally analysed using a S2 Picofox TXRF spectrometer (Bruker Nano GmbH, Berlin, Germany). The instrument was equipped with a Mo microfocus tube (30 W, 50 kV, 600 μA), a multilayer monochromator and a 30 mm^2^ XFlash^®^ silicon drift detector. All the samples were analysed for 1000 s of live time. Deconvolution and analysis of TXRF spectra were performed using SPECTRA 7^®^ software (Bruker Nano GmbH, Berlin, Germany). “Tomato leaves” (NIST 1573a) and “White cabbage” (BCR 679) certified reference materials were used to assess the accuracy of the method.

In order to map the elemental distribution in leaves, a benchtop μ-XRF spectrometer (M4 Tornado, Bruker Nano GmbH, Berlin, Germany) was used. Maps were acquired under near-vacuum conditions (20 mbar) using a Rh X-ray source (50 kV, 600 μA, 30 W) with polycapillary optics, and two 30 mm^2^ XFlash^®^ silicon drift detectors. Micro-focused X-ray maps were collected using 25 μm spot size, 10 µm step size and 10 ms pixel-acquisition time. In order to increase the signal to noise ratio (S/N), scanning was repeated 3 times and the relative spectra were averaged. The line-scan was acquired with a step size of 15 µm and a pixel-acquisition time of 1000 ms, repeating 10 times the acquisition. Maps were elaborated with ESPRIT software (Bruker Nano GmbH, Berlin, Germany, version 1.3.0.3273) and hyperspectral data were further processed by PyMca 5.1.3 (Solè et al. 2007) and Datamuncher (Alfeld and Janssens 2015) softwares.

**References**

Alfeld M, Janssens K (2015) Strategies for processing mega-pixel X-ray fluorescence hyperspectral data: a case study on a version of Caravaggio’s painting supper at Emmaus. J Anal Atom Spectrom 30:777–789. https://doi.org/10.1039/c4ja00387j

Bravin M (2014) ^©^CIRAD/RHIZOtest. <https://rhizotest.cirad.fr/en/the-rhizotest/methodology>. Accessed 6 July 2022

ISO 16198 (2015) Soil quality plant-based test to assess the environmental bioavailability of trace elements to plants.

Solé VA, Papillon E, Cotte M, Walter P, Susini J (2007) A multiplatform code for the analysis of energy-dispersive X-ray fluorescence spectra. Spectrochim Acta - Part B At Spectrosc 62:63–68. https://doi.org/10.1016/j.sab.2006.12.002

**Fig. S1** Protocol followed for the RHIZOtest experiments according to ISO 16198:2015 (images modified from <https://rhizotest.cirad.fr/en/the-rhizotest/methodology>). (a) Seed germination in hydroponics. (b) Seedling pre-growth period in hydroponics. (c) Plants at the end of the hydroponic pre-growth period. (d) Experiments with soil: the root mat is in contact for 7 days with the soil layer, physically (but not chemically) separated by a 30-µm mesh nylon membrane.

**Fig. S2** Root/shoot biomass ratio (R/S) of durum wheat plants after 7 d of contact with the unpolluted or polluted soil, unheated or heated at 300°C and 500°C. The R/S ratio of plants after the hydroponic pre-growth phase is also reported for comparison (Hydroponic). The vertical line on each bar indicates the standard deviation (n=5). No significant variation was found between plants grown on the same type of soil (unpolluted or polluted) at the three temperatures, according to one-way ANOVA (p < 0.05).
